# Pediatric Surgical Reentry Strategy Following the COVID-19 Pandemic: A Tiered and Balanced Approach

**DOI:** 10.1177/00031348211011125

**Published:** 2023-02

**Authors:** Ryan C. Pickens, Angela M. Kao, Mark A. Williams, Andrew C. Herman, Jeffrey S. Kneisl

**Affiliations:** 1 Department of Surgery, 2351 Atrium Health, Charlotte, NC, USA; 2 Levine Children’s Hospital, 2351 Atrium Health, Charlotte, NC, USA; 3 Surgical Care Division, 2351 Atrium Health, Charlotte, NC, USA; 4 Musculoskeletal Institute, 2351 Atrium Health, Charlotte, NC, USA; 5 Levine Cancer Institute, 2351 Atrium Health, Charlotte, NC, USA

**Keywords:** COVID-19, coronavirus, pandemic, elective surgery, pediatric surgery

## Abstract

**Background:**

In response to the COVID-19 pandemic, children’s hospitals across the country postponed elective surgery beginning in March 2020. As projective curves flattened, administrators and surgeons sought to develop strategies to safely resume non-emergent surgery. This article reviews challenges and solutions specific to a children’s hospital related to the resumption of elective pediatric surgeries. We present our tiered reentry approach for pediatric surgery as well as report early data for surgical volume and tracking COVID-19 cases during reentry.

**Methods:**

The experience of shutdown, protocol development, and early reentry of elective pediatric surgery are reported from Levine’s Children’s Hospital (LCH), a free-leaning children’s hospital in Charlotte, North Carolina. Data reported were obtained from de-identified hospital databases.

**Results:**

Pediatric surgery experienced a dramatic decrease in case volumes at LCH during the shutdown, variable by specialty. A tiered and balanced reentry strategy was implemented with steady resumption of elective surgery following strict pre-procedural screening and testing. Early outcomes showed a steady thorough fluctuating increase in elective case volumes without evidence of a surgery-associated positive spread through periprocedural tracking.

**Conclusion:**

Reentry of non-emergent pediatric surgical care requires unique considerations including the impact of COVID-19 on children, each children hospital structure and resources, and preventing undue delay in intervention for age- and disease-specific pediatric conditions. A carefully balanced strategy has been critical for safe reentry following the anticipated surge. Ongoing tracking of resource utilization, operative volumes, and testing results will remain vital as community spread continues to fluctuate across the country.

## Introduction

Since its emergence in December 2019, global spread of the novel coronavirus (SARS-CoV-2) and its associated clinical syndrome, coronavirus disease 2019 (COVID-19), has had an unprecedented impact on health and economy on a national and international level and has presented a myriad of unique challenges in the delivery of modern health care. Specifically, for the pediatric population, the COVID-19 pandemic has had a unique effect on the delivery of ongoing medical and surgical care. Although many of the early responses in health care have been documented, the specific impact on the surgical care of children and the response of children’s hospitals to the pandemic has been relatively underreported.

### Background

Early epidemiologic data suggested that SARS-CoV-2 affects children of all ages; however, compared to adults, children are affected less frequently with the majority being asymptomatic or reporting mild symptoms.^1,2^ The first pediatric COVID-19 case was reported in the United States on March 2, 2020, and preliminary data by the CDC reported 2572 pediatric COVID-19 cases representing 1.7% of confirmed COVID-19 cases occurring between February 12 and April 2, 2020.^3^ Despite the reduced burden of disease in children, the widespread shutdown of hospital-based services has had a significant impact on children’s hospitals as health care systems adapted in preparation for a potential surge of cases. The majority of children’s hospitals in the United States postponed elective and day pediatric surgery cases following The Centers for Medicare and Medicaid Services (CMS) consensus statement that recommended suspension of elective surgery in order to conserve hospital resources and reduce unnecessary exposure to patients and health care personnel.^4^ The Children’s Hospital Association and the American College of Surgeons (ACS) published elective case triage guidelines for surgical care during the COVID-19 pandemic, recommending only emergent and urgent pediatric surgery cases be performed.^5,6^ At the institutional level, additional modifications to normal surgical practice were reported during the shutdown such as implementing intubation protocols, restricting aerosolizing procedures such as laparoscopy and robotic surgery, and restructuring perioperative staffing using staggered teams to minimize exposure risk.^7^

### Reentry

Just as pediatric hospitals experienced a unique impact from the shutdown during the surge phase, similar challenges were faced with developing a strategic reentry plan for surgical care within the context of their unique patient population and resource environment. While guidelines for resuming elective surgery were jointly published by the ACS, American Society of Anesthesiologists, Association of periOperative Registered Nurses, and American Hospital Association, a similar set of guidelines specific to resuming pediatric surgical cases at children’s hospitals were not clearly outlined by pediatric surgical societies, with many deferring to the adult guidelines.^8^ Recommendations from the Children’s Hospital Association and the American Pediatric Surgical Association included considerations for expanding non-emergent operations in order to reduce the risk of hospital admission or prolonged hospital stay which could increase exposure risk and resource utilization.^9,10^ The variability in pediatric surgical guidelines likely reflects the wide range of impact across children’s hospitals and the rapidly evolving understanding of the risk of exposure vs. the delay in surgical care. In light of the complexity of this decision-making, the authors present the experience of their institution to highlight the unique considerations many children’s hospitals faced and provide a tiered approach for resumption of non-emergent essential pediatric surgical care.

## Levine Children’s Hospital

Levine Children’s Hospital (LCH) is a 234-bed free-leaning children’s hospital located on the campus of Atrium Health Carolinas Medical Center in Charlotte, North Carolina, part of a 40-hospital system with 900 care locations across North Carolina, South Carolina, and Georgia. Levine Children’s Hospital supports an 85-bed Level IV neonatal ICU (NICU) and 20-bed combined pediatric/cardiovascular ICU, as well as a 13-bed inpatient rehabilitation unit and provides a full spectrum of pediatric services including a Level I pediatric trauma center, extracorporeal membrane oxygenation, cardiac surgery, neurosurgery, and a dedicated pediatric emergency department. All surgical patients at LCH are managed with dedicated pediatric anesthesia. Levine Children’s Hospital shares operative resources with the adult hospital but maintains 4 general and 2 cardiac pediatric operating rooms (OR) with additional dedicated ORs in the ambulatory surgery center.

## Strategically Plan for Reentry

**Figure 1.**
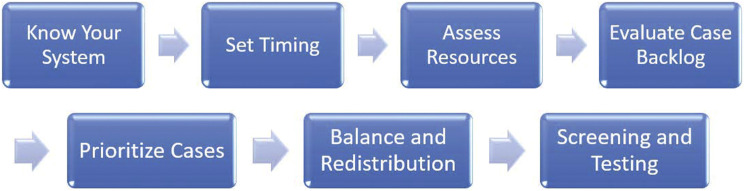
Critical steps for Reentry at Levine Children’s Hospital.

As the trajectory of COVID-19 cases began to stabilize in the region surrounding the authors’ institution, the leadership of LCH along with a pediatric-specific COVID-19 Surge and Re-entry Team set about drafting a strategic plan for safely resuming electively timed, appropriately indicated essential pediatric surgery. Our institution identified a set of critical principles necessary for safe reentry, each reflecting the complex shared decision-making required to ensure a balanced approach within the context of the continually evolving pandemic response (Figure 1). These included ensuring a manageable trend in COVID-19 cases prior to reentry timing, adequate capacity and resources to ensure patient and provider safety with the increased patient volume, cataloging the case backlog, developing a strategic approach for case prioritization and scheduling, and installing a rigorous perioperative screening and testing protocol. Each principle was addressed based on the unique considerations of our children’s hospital and the impact of COVID-19 within the community it serves.

### Knowing the System

The foundation for structuring a reentry protocol was understanding the relationship of the children’s hospital within our unique health care system and region. As a free-leaning children’s hospital, decisions to resume surgical care would have ramifications on the hospital’s primary shared resources including personal protective equipment (PPE) supply and distribution, blood bank and laboratory services, diagnostic and interventional radiology support, and recalling furloughed staff members. A clear pipeline of regular communication was immediately established with leadership teams to ensure that the shared responsibility of balancing the needs of all patients and health care workers was preserved with each step toward reentry. As the rollout process began, an accruing assessment of resource supply and demand as well as divisional protocols for safe care (ie, testing required before non-emergent radiology studies) was maintained.

### Timing of Reentry

As regional trends of coronavirus cases varied widely across the country, safely resuming non-emergent pediatric surgical care was implemented in alignment with local and national health authority recommendations. Timing of reentry was initially based on the ACS and CMS guidelines of a sustained reduction in the rate of new COVID-19 cases.^11-13^ However, due to the extension and flattening of the predicted peak in the region’s population, resumption of electively scheduled, essential surgery was determined to be necessary and safe after the rate of COVID-19 cases was approaching its anticipated peak. Adult surgical reentry was, therefore, scheduled to begin on May 11, 2020. Due to the relatively lower impact of COVID-19 cases on the pediatric population and the availability of resources in LCH as a result of the shutdown, the pediatric surgery was selected to be the torchbearer and cases were scheduled to begin 1 week ahead on May 4, 2020.

### Assessment of Resources

The redistribution of resources (including, but not limited to, OR availability, hospital/ICU beds, ventilators, PPE, and personnel) from many children’s hospitals to support the anticipated surge of adult COVID-19 patients presented an additional unique challenge. The unique relationship between each children hospital and its parent health system necessarily impacted the pathway for reentry in different ways. In areas of the country greatly affected by COVID-19 known as “hotspots,” children’s hospitals mobilized resources such as ventilators and PPE to adult hospitals facing critical shortages, while others converted children’s hospital units to care for adult COVID-19 patients.^5^ Similarly, redeployment of personnel, such as anesthesiologists, pediatric critical care intensivists, and nurses, to adult intensive care units reduced pediatric surgical resources. In many cases, reallocation of resources such as ventilators, nursing staff, and PPE back toward pediatric hospitals to safely support the ramping up of pediatric surgical care was a shared decision with the adult hospital to ensure resources remained adequate across the system for the current environment and the potential for a second surge.^6^

Our Chief Surgical Officer (JSK) identified 8 critical decision factors that served as the pillars for how to balance the resource assessment for a surgical reentry strategy (Table 1). Each resource was assessed independently and within the context of the greater health care system as each decision factor potentially leads to resource strain in multiple areas. Under the leadership of the Surge and Re-entry Team, a management template was created, allowing for sufficient flexibility for future modifications by each surgical service.

**Table 1.** List of Critical Decision Factors With Challenges and Proposals.

Critical decision factor	Questions/challenges	Action/proposed solution
Patients	Prioritize backlogged vs. new?	Prioritize healthy, outpatient
	Plan for preoperative testing/screening?	Screen and test all patients prior to surgery
	Comorbidities and risk?	Limit patients requiring ICU, transfusion, known need for SNF/rehab postoperatively
	Require ICU post-op?	Surgeons proactive scheduling
	Blood requirements?	
Surgeons	Distribution of OR time with amount of backlog?	Balance OR time by previous block time and case volume
	Quantity and quality by location?	Encourage appropriate case location (outpatient cases in outpatient/ASC settings)
	Availability for evening and weekends if able to expand OR utilization?	Ascertain availability of extending hours
	Plan for testing/screening?	Continue routine screening- test symptomatic
Anesthesia	MDs and CRNAs available?	Await end to redeployment
	Redeployment?	Increase anesthesia coverage proportional to ORs running and increased hours
	Coverage of increased case numbers?	Continue routine screening- test symptomatic
	Pre-operative screening clinic?	
	Plan for testing/screening? (High risk)	
Staffing	Availability of RNs, scrub techs, OR assistants?	Strategic hiring new positions
	Effect of redeployment on staffing?	Await end to re-deployment
	Flexibility to increase hours and staff appropriately?	Communication with administration for increased OR utilization
	Plan for testing/screening?	Continue routine screening- test symptomatic
Operating rooms	Inpatient- strategic ramp up?	Active communication with administration regarding numbers
	ASC/HOPD- exposure to COVID-19?	Non-COVID-19 care facilities
	COVID-19 safe facilities?	
Equipment- capital/technology	Appropriate use of virtual technology?	Utilize virtual visit for pre-op screening
	Scheduling platform for patients/surgeons?	Increase access to online scheduling platform
	Adequate PPE for surgeons, anesthesia, staff?	Central evaluation of OR assets
		Confirm adequate supply of PPE
Bed capacity	Hospital bed utilization?	Ensure stable bed utilization
	ICU bed limit?	Limit elective cases with known or suspected need for ICU postoperatively
Infrastructure	Sterile processing capacity for OR flow?	Confirm ability to accommodate increased case numbers and turnover
	Supply chain disruption for implants/supplies?	Confirm maintained supply chain and adjust for disruptions

Abbreviations: ICU, intensive care unit; SNF, skilled nursing facility; ASC, ambulatory surgery center; HOPD, hospital outpatient department; PPE, personal protective equipment.

### Assessment of Case Backlog

**Figure 2.**
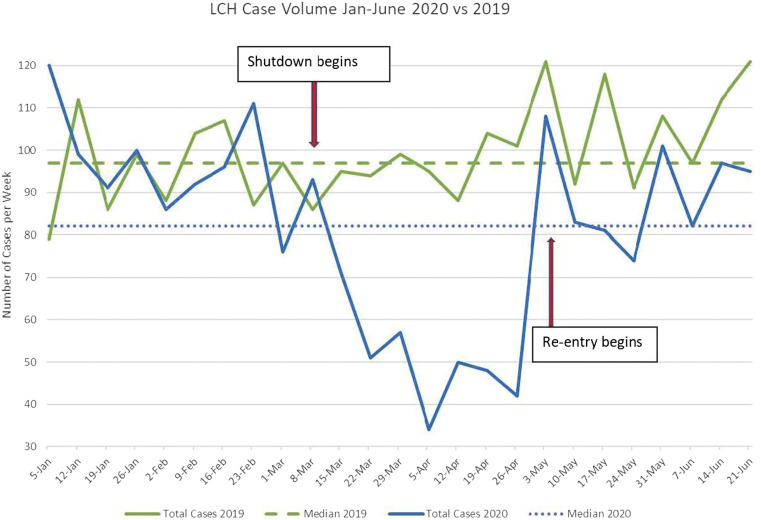
Trend of surgical case volume at Levine Children’s Hospital in 2020 compared to 2019.

**Figure 3.**
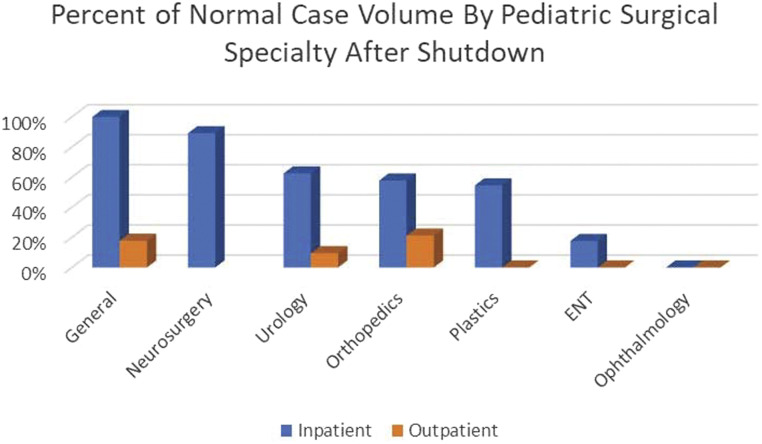
Percent of normal case volume during shutdown by surgical specialty (March 15 through May 2, 2020 compared to 2019).

Following a comprehensive assessment of resources, evaluating the volume of cases suspended during the shutdown of elective surgery was the next critical step to determining the appropriate reallocation of these resources. Prior to COVID-19 restrictions, operating room utilization at LCH was at near maximum capacity with an average case volume of more than 420 cases per month in January and February 2020 immediately preceding the cessation of elective surgery on March 17, 2020. During the shutdown, pediatric surgery at LCH decreased to only 55% of the weekly case volume compared to the preceding months (January/February 2020). Similarly, compared to an identical time frame in the previous calendar year (March/April 2019), pediatric surgical case volume decreased to 53% (Figure 2). This resulted in a backlog of approximately 400 surgical cases across all pediatric subspecialties. The greatest impact was expectedly on the ambulatory surgery center which experienced a 90% decrease in operations, while inpatient operations were reduced by 18%. Pediatric surgical subspecialties experienced variable impact in both the inpatient and outpatient settings (Figure 3). These data were used to assess needs for reallocating resources and tracking the ability of each program to catch up over the subsequent months.

### Prioritization of Surgical Cases

Appropriate prioritization of surgical cases is critical to the development of a tiered approach for resuming elective surgery. For surgeries that can be performed in a non-emergent or nonurgent fashion, it is important to balance both need and benefit to the patient.^14^ During the prioritization process, compiling a list of previously canceled/postponed cases and calculating the required resources and staff for each may better enable objective assessment of the backlog of surgical cases and new cases needing to be scheduled.^8,12^ At our institution, our tiered approach for reentry and resuming pre-COVID-19 surgical volume was to initially target the healthiest patients with the highest need and potential benefit from their procedure.

Pediatric-specific considerations that further added to the complexity of case prioritization include the impact of delaying surgery on age- and growth-dependent interventions for congenital conditions.^10^ Within the pediatric population, many operations require careful timing within a patient’s growth and development and substantial delay may hold significant consequences. Repair of congenital malformations inherently balance timing between ensuring an adequate size of the child with reestablishing function as early as possible. Musculoskeletal malformations and injuries both require careful consideration of development and healing for the timing of surgical intervention. Similarly, a delay in repair of gastrointestinal anomalies may impact a child’s nutritional support and functional ability during a critical development period.

In light of the relatively lower morbidity and mortality of COVID-19 within the pediatric population, the risks of delaying necessary surgical interventions in children was considered even more strongly. Thus, pediatric patients were selected to pilot the first phase of surgical reentry. A tiering system was developed to assist surgeons and surgical teams with case prioritization (Table 2). Given the variable impact on surgical volume and case backlog across the different pediatric subspecialties, each surgical service line was encouraged to tailor the reentry template to accommodate their specific patient population.

**Table 2.** Levine Children’s Hospital Guideline for Tiered Prioritization of Pediatric Surgical Cases.

Atrium health Levine children’s tiering of surgeries					
Measure	Tier 1	Tier 2	Tier 3	Tier 4	Tier 5
ASA level	ASA 1	ASA I-II	ASA I-III	All	All
Special considerations/implications if delayed	ODS	1. QOL issues	1. QOL issues	All cases	All cases
		2. Nutritional implications	2. Nutritional implications		
		3. Developmental implications for staging	3. Developmental implications for staging		
		4. Delay risking complicated approach, technique, or recover	4. Delay risking complicated approach, technique, or recover		
Comorbidity	Minimal	Minimal	Moderate	All	All
ICU risk	None	None	Minimal	Moderate	Likely
Expected total LOS	<1 day	<2 days	<4 days	4 ≥ days	4 ≥ days
Age	>2 months PCA	>2 months PCA	All	All	All

Abbreviations: ASA, American Society of Anesthesiologists; ODS, one-day surgery; QOL, quality of life; LOS, length of stay; PCA, postconception age.

### Balance and Redistribution of Cases

Delivering safe, uncompromised patient care while maintaining flexibility in the event of a second potential surge remained the highest priority in our coordinated effort to resume surgical cases. Redistribution of surgical cases according to the patient and procedure allowed for healthy patients with acceptable case level acuity to be preferentially directed to ambulatory surgical centers, reserving hospital outpatient and inpatient settings for patients that necessitated higher levels of care. With these considerations, the health care system initiated the reentry pilot phase with outpatient cases in the ambulatory surgery center and hospital outpatient department settings 1 week prior to resuming elective inpatient cases. A limited volume of pediatric cases was first scheduled in the hospital outpatient surgical center 1 week prior to resuming elective cases in the inpatient OR. The projected timeline was to resume 100% of pre-COVID-19 caseload volume within 6 weeks with a further ramp up to 120% until the backlog of cases has been addressed.

### Screening and Testing for COVID-19

Screening and testing practices have been essential for limiting spread and avoiding potential exposures during the surge phase of COVID-19. However, perioperative screening and testing is a constantly moving target as rates of asymptomatic or minimally symptomatic carriers continue to rise and laboratory testing technology evolves rapidly.

While children are affected by COVID-19 less frequently and less severely than adults, this may lead to a lower rate of testing in children in the community, thus underestimating the true burden of disease and raising the potential for children being asymptomatic carriers of transmission. Importantly, the severity of illness widely varies by age with 1 study of pediatric COVID-19 patients reporting severe or critical symptoms in 10.6% of infants less than 1 year of age.^1^ Additionally, early data in the adult population demonstrated worse outcomes after surgery for COVID-positive patients.^15^ Consequently the decision was made to screen and test all children prior to elective surgery at LCH to promote the safety of the patients, other children, and health care providers.

**Figure 4.**
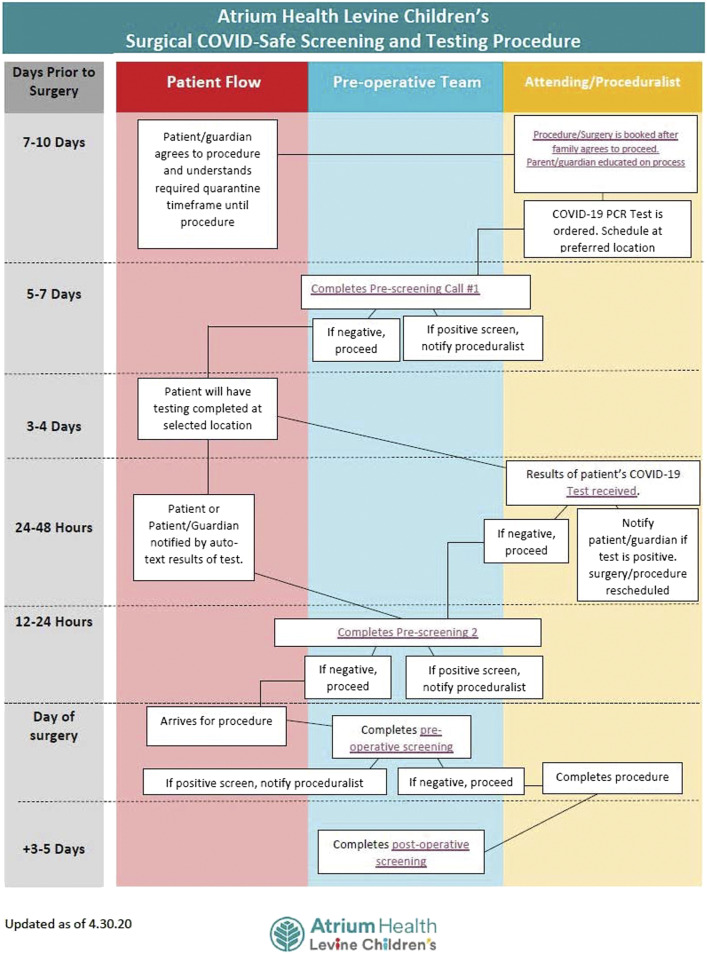
COVID-19 preoperative screening and testing procedure for elective pediatric surgery at Levine Children’s Hospital.

**Figure 5.**
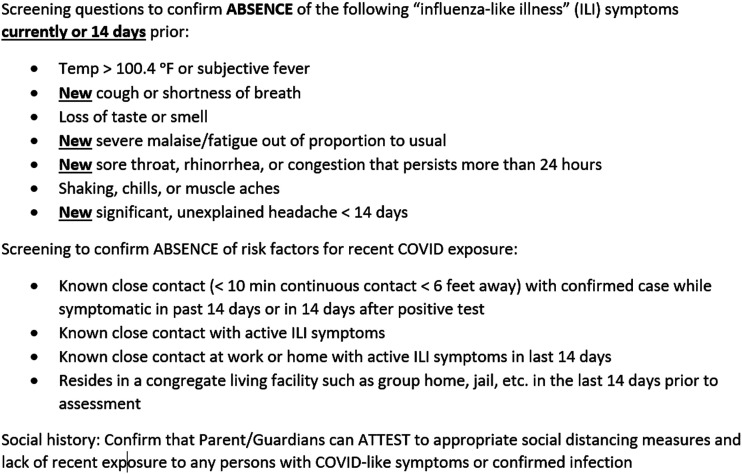
COVID-19 screening questionnaire for elective pediatric surgery at Levine Children’s Hospital.

Based on the best available evidence and consensus within the LCH Surge and Re-entry Team, a standardized surgical COVID-19 safe screening and testing procedure was established for all elective surgery at LCH (Figure 4). All patients are screened using a standardized questionnaire (Figure 5) followed by PCR testing. On the day of surgery, all patients are again screened for symptoms and/or exposure and verified to be afebrile (<100.4 °F) before entering the preoperative area. Finally, patients are again contacted for screening on postoperative day 3-5 to track potential missed exposures.

## Early Outcomes After Reentry at LCH

### Surgical Volume

Once the reentry process was initiated, intentional tracking of surgical case volumes, pre-procedural COVID-19 screening and testing results, as well as continued trends of COVID-19 within the community were essential to ensuring the safety of providers and patients. In the first 8 weeks of reentry (May 4-June 21), 721 pediatric operations were performed at LCH. As shown in Figure 2, the early surgical case volumes after reentry at the authors’ institution demonstrated an unanticipated trend. Although the predicted trajectory after reentry was to be a steady escalation of case volume, the actual case volumes immediately spiked during week 1 but subsequently slowed down over the next few weeks of reentry, despite efforts to ramp up OR availability and case scheduling. Various explanations have been reported by local surgeons anecdotally including parents electing to delay operations due to concerns about their children receiving COVID-19 testing or being hospitalized, as well as a reduced volume of referrals from primary care physicians who are still returning to their pre-COVID-19 patient volumes. Ongoing data collection is now being implemented to objectively document these reasons to understand how to address these concerns to ensure that children’s surgery is not unduly delayed when medically important. With increased operating room availability, surgical divisions have also escalated through their prioritization tiers to resume surgery for more at-risk children and case volume seems to be increasing again, although not yet consistently back to pre-COVID-19 volume.

### Pre-Procedural Screening/Testing

**Figure 6.**
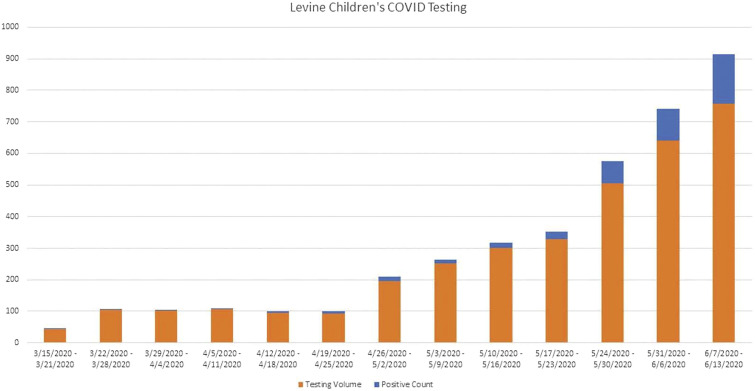
Trend of COVID-19 testing results at Levine Children’s Hospital.

Figure 6 shows the trend of COVID-19 testing and positive cases performed throughout LCH since the shutdown through the first 8 weeks of reentry. The initial positive test percentage remained low during the first few weeks of reentry; however, as community spread continues after staged lifting of social restrictions, the volume of positive tests seems to be increasing, although this is also within the context of significantly increased outpatient testing for symptomatic children as well. To date, the number of actual pre-procedural COVID-19 screening and testing results returning positive at the authors’ institution has remained only a small fraction of these positive tests and thus have not prohibited advancing to higher tiers of case prioritization to address the backlog of non-emergent surgical care.

### Post-Procedural Screening

Post-procedural screening is also critical to track any incidence of potential hospital-acquired transmission of COVID-19 and identify the source of transmission as early as possible. Through the first 6 weeks of reentry, 7 patients (1.3%) have failed post-surgery screening follow-up with influenza-like illness symptoms or concern for contact with other COVID-19-positive individuals. Of these, 1 patient met criteria for COVID-19 testing which was negative. All others were instructed to quarantine and had self-limiting symptoms.

## Discussion

In response to the evolving COVID-19 pandemic, health care facilities and surgical practices faced numerous challenges as they rapidly adapted to dramatic reductions in surgical volumes with cancellation of non-emergent/urgent procedures and disruption of perioperative resources and staff. Planning for surgical reentry presented additional challenges for children’s hospitals, particularly those incorporated within a larger health care system that may share critical perioperative resources with adult hospitals. Navigating the logistics of surgical reentry at such larger institutions can be complex and often requires balancing the needs of multiple surgical service lines and consideration of numerous factors. The described process and template, in combination with a decisive leadership team, were fundamental in the development and implementation of a tiered approach to surgical reentry at the authors’ institution. As children’s hospitals return to elective surgery and address significant backlogs, it is paramount to select the appropriate pace, patients, and location for reentry and ensure adequate resources are available. Such decisions should not be made by health care institutions in a vacuum, but rather in conjunction with regional government and public health departments in a balanced and collaborative manner, so as not to jeopardize the health of the local community. However, as community and hospital cases of COVID-19 begin to rise again following recently relaxed social distancing restrictions across the country, readiness to scale back surgical reentry in an expeditious time frame must be continually reevaluated.
